# Investigation of the Mechanical Response of the Foot Structure Considering Push-Off Angles in Speed Skating

**DOI:** 10.3390/bioengineering10101218

**Published:** 2023-10-18

**Authors:** Haichun Wang, Yusen Wu, Jingxi Liu, Xiaolan Zhu

**Affiliations:** 1 Sport Science School, Beijing Sport University, Beijing 100084, China; 2 School of Naval Architecture and Ocean Engineering, Huazhong University of Science and Technology, Wuhan 430074, China

**Keywords:** speed skating, push-off angle, finite element model, foot, biomechanics

## Abstract

The push-off angle is an important factor affecting speed-skating performance. However, quantitative evidence for the relationship between the push-off angle and foot injury is incomplete. This study aimed to establish a three-dimensional (3D) finite element model (FEM) and investigate the mechanical responses of foot structures to stress and strain to explore the relationship between injury and movement. A 3D FEM was reconstructed using CT and 3D scan data and validated by comparing the FEM-predicted and in vivo measurement data in the balanced standing state. A push-off angle obtained from a video of a champion was loaded into the FEM. The error rates of validation were less than 10%. With a decrease in the push-off angle, the stress on the metatarsal increased; the stress on the talus, ankle joint cartilage and plantar fascia decreased, as did the strain on the ankle joint cartilage and plantar fascia. The FEM was considered reasonable. Not all foot structures had an increased risk of injury with a decrease in the push-off angle from 70° to 42°. The FEM established in this study provides a possibility for further determining and quantifying the relationship between foot injury and skating technique.

## 1. Introduction

Speed skating is a competitive sport in the Winter Olympic Games [1]. Performance in speed skating is related to the push-off angle, the speed of skating, the push-off force and the time of skating [2,3]. In the process of speed skating, the ice reaction force of the push-off force is divided into a vertical upward force and a horizontal force perpendicular to the forward direction [4]. The acceleration generated by the component perpendicular to the forward direction combines with the original forward-direction acceleration to produce new forward direction speed when the athlete’s legs alternate [5,6,7]. Noordhof D. A. et al. found that the speed of speed skating is closely related to the push-off angle [8,9]. The push-off angle is generally defined as the angle between the reaction force of the push-off force and the surface of the ice [4], so the efficiency of pushing off of the ice is improved when the push-off angle is smaller [2,4,10]. Studies have shown that for every 1° decrease in the push-off angle, speed increases by 0.011 m/s [11]. In speed skating at a speed above 60 km/h [12], the difference between first and second place often lies between 0.01 and 0.02 s [13]. As a result, the change in the push-off angle may have a direct impact on an athlete’s performance and their further success in competitions.

It has been suggested that a smaller push-off angle may be conducive to increasing the skating speed [8,9]. However, an overly small push-off angle may increase the risk of foot injury [14,15]. Studies have determined that 64.2% of athletes are injured at least once a year [16], with foot and ankle injuries accounting for about 14% of all cases [17]. And among the causes, long-term excessive load, abnormal force and fatigue due to the use of foot structures are considered the most common injuries and are manifested as stress and strain concentrations or excessive stress and strain [18,19,20]. Consequently, athletes fail to participate in training and competition immediately or for a long time, and suffering from sports injuries can even have irreversible impacts on their sports careers. Given the studies above, a scientific assessment of the risk of foot injury is beneficial to coaches and athletes so they may adopt reasonable technical movements and reduce the risks of injury. However, despite extensive retrospective, investigative research, quantitative research on speed-skating injuries is scarce [16,21].

Inadequate movement during speed skating is an important factor causing athlete foot injuries. It has been shown that speed skating is a complex sport that relies on mechanical factors and the intricate coordination of neural substrates to regulate muscle contractions, balance and precise movements [22,23,24]. Although recent studies have shown that the push-off angle is related to the risk of foot injury to a certain extent, the movement mechanics of the internal structure of the foot cannot be obtained from the rapid speed-skating process due to limited experimental conditions [3]. In addition, it is difficult to comprehensively and directly explain the source of the risk of injury and accurately locate the injury site. It is worth mentioning that the finite element method, which applies a method of numerical calculation according to the principle of approximate segmentation, provides a possibility of solving the above problems. It can not only analyze and predict load changes in the internal structure of the human body under certain working conditions by reconstructing a three-dimensional (3D) finite element model (FEM) but can also provide numerical information about stress and strain. At present, the finite element method has been successfully applied in predicting the risk of foot injury in ice hockey [25], evaluating the effect of the hardness of the midsoles of running shoes on plantar fasciitis [26] and the design of badminton insoles [27].

Therefore, this study aimed to establish a 3D FEM of a foot and a speed skate, simulate and calculate the speed-skating movement and investigate the effects of different push-off angles on the mechanical responses of foot structures during speed skating. It can provide a basis for further research on the quantitative relationship between push-off angle and foot injury, as well as the clarification of the specific injury site on the foot.

## 2. Materials and Methods

**Figure 1:**
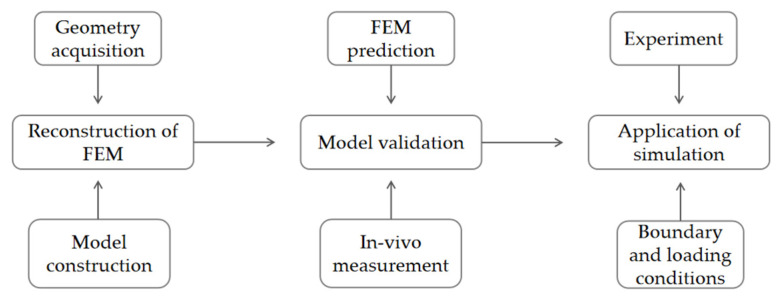
The content of this research study.

The design of this study involved the reconstruction of a 3D FEM, assigning properties to the material, verifying the effectiveness of the model and then obtaining the loading conditions of the push-off angle through experiments, to conduct an FEM simulation and obtain stress and strain data for the foot structure (Figure 1).

### 2.1. Subject and Speed Skate

The FEM of a foot and a speed skate was based on the right foot of a 25-year-old male athlete who read and signed an informed consent form (height, 167 cm; weight, 64 kg; length of foot, 26 cm; width of foot, 7.5 cm; no history of foot disease or deformity) and a speed skate that was provided by the manufacturer. The material of the upper of the speed skate, lining and skate blade were straw mat mirror space leather, polymerized styrene butadiene rubber (SBR) memory sponge and carbon fiber, respectively. The hardness of the blade was HRC62-64. The research protocol was approved by the ethics committee of Beijing Sport University (2021114H). 

### 2.2. The Reconstruction of the FEM

#### 2.2.1. Geometry Acquisition

Imaging data of the right foot in a weightless, neutral position, from the sole of the right foot to the ankle joint, were obtained using a computed tomography scanner (Philips 16-row spiral CT machine, Amsterdam, The Netherlands) with a layer thickness of 0.67 mm. The CT values of the bone and the soft tissue were 180-3071 and -45-3071, respectively. The geometry of the foot, including the bone and tissue, was reconstructed using MIMICS19.0 medical software (Materialise, Leuven, Belgium) by filtering the data of the scanned foot and optimizing the plane images of the bone and soft tissue of the foot. Point cloud data of the vamp, butt and blade of the speed skate were obtained using a 3D laser scanner (Creaform handyscan 700, Lévis, QC, Canada) with a scanning resolution of 0.8 mm. Then, the foot and the speed skate geometry model were processed using Geomagic Studio 2013 (Raindrop Geomagic Inc., The Triangle Development, NC, USA). On the basis of not affecting the subsequent simulation calculation results, the shoelace and zipper on the upper surface of the speed skate were simplified to improve the calculation efficiency.

#### 2.2.2. Model Construction

The scanned data of the foot and the speed skate were imported into ANSYS WORKBENCH 20 R2 (Swanson Analysis, Houston, PA, USA) for assembly. The upper lining was built using a Boolean computing function between the soft tissue and the speed skate, and the supporting ground, the surface of the ice (60 cm × 30 cm × 10 cm) and an EVA cushion (60 cm × 30 cm × 0.2 cm) were established in this software, respectively. The plantar fascia was established using a LINK180 unit, the ligaments were established using a spring unit and the model was meshed using a tetrahedral unit.

For contact between components, bounding contacts were used between the bones and the interior surfaces of the soft tissue, ligaments and articular cartilage, and frictionless contacts were used between the bones and joint surfaces. In addition, the soft tissue of the foot and the speed skate were set to have friction contact with a friction coefficient of 0.6 [28]. Considering the complexity of biological tissues and computational efficiency, the bones and articular cartilage were considered homogeneous isotropic and linear elastic material in this study. The relationship of the linear elastic model was expressed as follows:

(1) $$\sigma_{ij}=E_{ijkl}\varepsilon_{kl}$$

In the above equation, $\sigma_{ij}$ is the component of the stress tensor, $E_{ijkl}$ is the component of the elasticity tensor, and $\varepsilon_{kl}$ is the component of the strain tensor. For isotropic bodies, $E_{ijkl}$ has two independent components, and the constitutive relation can be further simplified as follows:

(2) $$\sigma_{11}=\frac{\mu E}{\left(1+\mu \right)\left(1-2\mu \right)}\left(\varepsilon_{11}+\varepsilon_{22}+\varepsilon_{33}\right)+{2G\varepsilon }_{11}$$

(3) $$\sigma_{22}=\frac{\mu E}{\left(1+\mu \right)\left(1-2\mu \right)}\left(\varepsilon_{11}+\varepsilon_{22}+\varepsilon_{33}\right)+{2G\varepsilon }_{22}$$

(4) $$\sigma_{33}=\frac{\mu E}{\left(1+\mu \right)\left(1-2\mu \right)}\left(\varepsilon_{11}+\varepsilon_{22}+\varepsilon_{33}\right)+{2G\varepsilon }_{33}$$

In the above equation, *E* is the elastic modulus, *G* is the shear modulus and μ is Poisson’s ratio.

**Figure 2:**
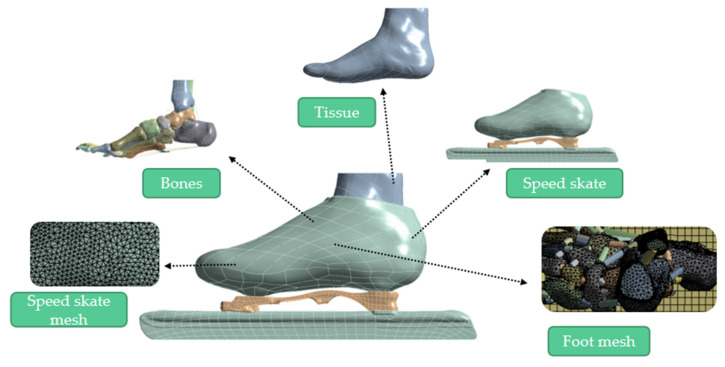
Finite element model of a foot and a speed skate.

In this study, material parameters of the foot in the model, which were idealized as homogeneous, isotropic and linear elastic, were obtained from literature [25,29,30], and the parameters for the speed skate and the surface of the ice were provided by the manufacturer to improve the accuracy of the calculation (Table 1). There were 30 bones, 29 pieces of cartilage, 109 ligaments, 5 plantar fascias, 1 encapsulated soft tissue and 1 speed skate in the FEM, generating a total of 1,310,568 nodes and 842,670 elements (Figure 2).

### 2.3. Model Validation

It is an essential step to test the model validation in the implementation of a simulation. It is generally accepted that the error rate between the measured and the simulated results is used to verify the model. The smaller the error rate, the more effective the model is. The FEM was validated by comparing the contact pressure peak, contact area and the distribution of pressure on the plantar and the bottom of blade between the FEM’s prediction and in vivo measurements obtained during balanced standing. Validation data were obtained using a Pedar plantar pressure insole (PEDAR-X; Novel, Inc., Munich, Germany) and a Novel emed-x plantar pressure test plate (Novel, Munich, Germany) simultaneously when the subject was standing in a natural, balanced state on speed skate, with a sampling frequency of 100 Hz. In order to avoid damage from the speed skate to the test plate, a 2 mm layer of an ethylene vinyl acetate copolymer (EVA) cushion was placed between the speed skate and the test plate. The subject was the foot model provider, and he was tested three times according to the above requirements to ensure the validation of the collected data.

In the FEM, a cushion of a 2 mm layer of EVA was placed between the speed skate and the ground, and the friction coefficient between the speed skate and the EVA cushion was 0.6. The distal tibia and fibula bones and soft tissue were fixed. The ground displacement was constrained in four directions, and the vertical ground reaction force of the foot was 320 N (half of the body weight), obtained via the vertical upward displacement from the ground [25]. In this study, the Gastrocnemius muscle showed more significant activity during balanced standing [31], and its strength was only considered to be the Achilles tendon force, ignoring other muscle forces in the foot. As a result, the Achilles tendon force was loaded to 160 N at the attachment point of the Achilles tendon via the node force [32].

### 2.4. Application of Simulation

The load and boundary conditions in this study were that the push-off angles obtained in the experiment were loaded into the FEM. Then, skating action modeling with different push-off angles was carried out to obtain the stress and strain values of the foot structure.

#### 2.4.1. Push-Off Angle Experiment

**Figure 3:**
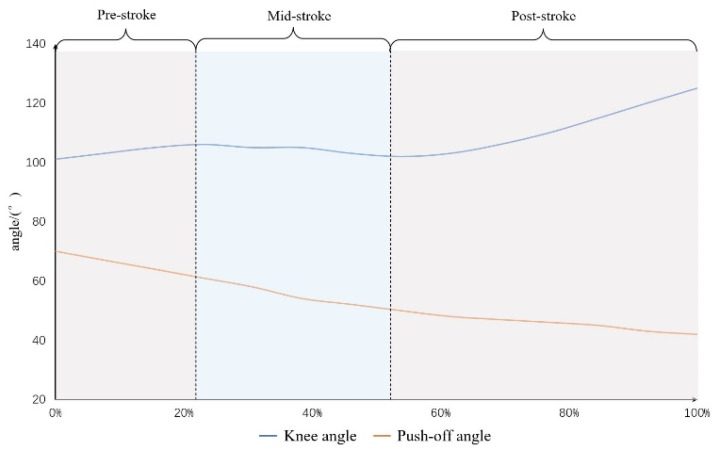
Speed-skating stages per stroke.

The champion of the 2019–2020 National Speed Skating Men’s 500 m Championship was selected as the research object in this study. The athlete’s speed-skating movement during the first lap of the race in the straight stage was captured using 4 high-definition video cameras (SONY FDR-AX700, Sony group corporation, Tokyo, Japan) with a resolution of 1920 × 1080. Two cameras were located on the side of the track, and the other two cameras were on the front of the track. The shooting range was 76 m from the starting point. With a shooting frequency of 60 frames/s and a shutter speed of 1/500 s, the angle of the main optical axis of each of the two cameras was about 100°. The video was analyzed using an artificial intelligence system for the automatic capture of human motions (Beijing Sport University in cooperation with Dalian RuiDong Technology Co., Beijing, China). The two-dimensional coordinates of 21 joints of the human body, obtained using two cameras, were obtained by the system. Then, the analytical data were synthesized into 3D coordinates on a geodetic coordinate system using the direct linear transformation method, and the resolved data were filtered using the Butterworth low-pass filtering method with a cut-off frequency of 10 Hz. In this study, only the push-off angle in the frontal plane and the knee angle in the sagittal plane were considered. The push-off angle was defined as the angle between the right leg (the line connecting the center point of the hip–ankle joint) and the ground at the moment before the speed skate hit the ground, and the knee angle referred to the angle between the center points of the hip, knee and ankle joints in the middle stage of right leg support [11]. Finally, the push-off angle and the knee angle of the athlete in the straight stage ranged from 42° to 70° and 101° to 125°, respectively. Since the knee angle was an important factor for evaluating the speed-skating technique of athlete [2,10,33,34], the speed-skating movement was divided into three stages (pre-stroke, mid-stroke and post-stroke stages) based on the changes in the athlete’s knee angle from extension, then flexion and then extension in this study [34]. The push-off angle ranges for the three stages were 70°–63° in the pre-stroke stage, 63°–49° in the mid-stroke stage and 49°–42° in the post-stroke stage (Figure 3).

#### 2.4.2. Boundary and Loading Conditions

**Figure 4:**
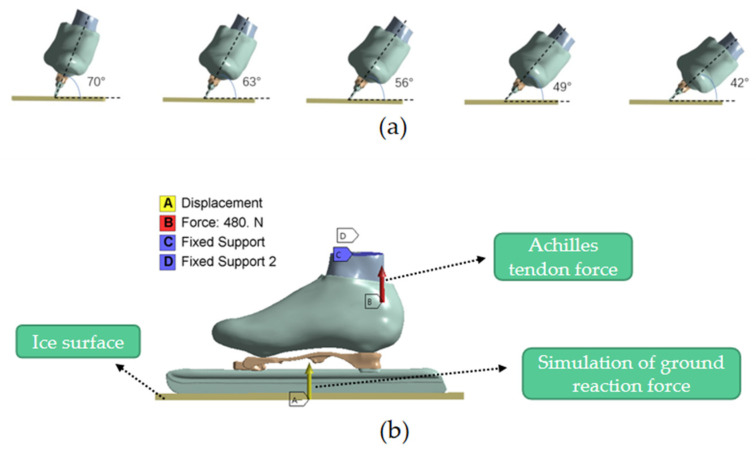
Boundary and loading conditions, (**a**) five push-off angles (**b**) and the push-off during skating.

Speed-skating strokes were simulated in the FEM with push-off angles of 70°, 63°, 56°, 49° and 42°, respectively (Figure 4a). During the simulation, the bones and soft tissues of the distal tibia and fibula were fixed. Only the forces along the push-off angle were considered as the push-off forces of the ground reaction forces. Meanwhile, only the Gastrocnemius muscle’s strength was considered, and the strength of the other muscles of the foot during speed skating was ignored in this study. The ice surface displacement was constrained in four directions. The ground reaction force of the foot was 640 N (the body weight), obtained via the vertical upward displacement from the surface of the ice with a friction coefficient of 0.003 between the surface of the ice and the speed skate [28,35] (Figure 4b). The force of the Achilles tendon was set at 480 N (three-quarters of the body weight) applied to the attachment point of the Achilles tendon and the calcaneus. The stress and strain data for the calcaneus, talus, metatarsal, ankle joint cartilage, subtalar joint cartilage and plantar fascia were obtained by simulating different push-off angles in the FEM.

## 3. Results

### 3.1. Model Validation

**Figure 5:**
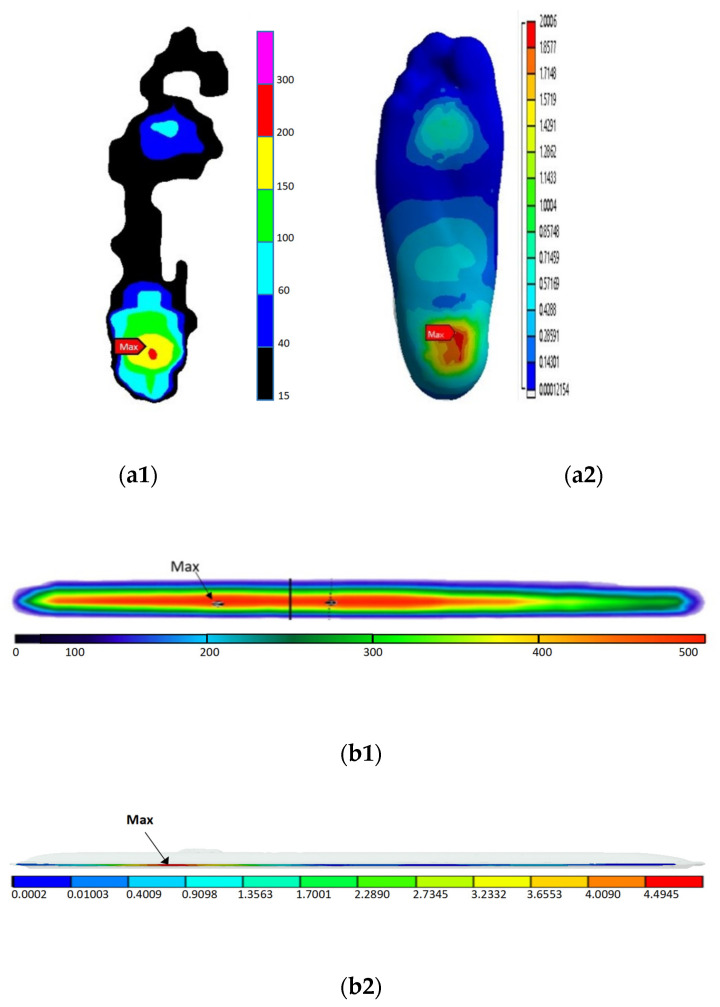
The verification results. (**a1**) The measured plantar distribution; (**a2**) the simulated plantar distribution; (**b1**) the measured distribution of the bottom of the blade; (**b2**) the simulated distribution of the bottom of the blade.

The peaks of plantar pressure were mainly located in the heel and measured using the plantar pressure insole and the FEM. According to the distribution of plantar pressure using the FEM prediction and in vivo measurements, the stresses, which were similar, were mainly distributed in the forefoot and heel (Figure 5). The error value of the peak of the plantar pressure was 20 kPa, and the error rate was 9.0%. The error value of the plantar contact area was 8.1 cm^2^, and the error rate was 7.6% (Table 2).

The peaks of pressure at the bottom of the blade, which were measured via the plantar pressure test plate and the FEM, were both concentrated on the back side of the blade bottom, which was below the heel. With pressure values that were smaller than in other places at the tip and tail of the blade, the stress distribution at the bottom of the blade was consistent (Figure 5). The error value of the peak pressure at the bottom of the blade was 47 kPa, and the error rate was 9.5%. The error value of the contact area of the bottom of the blade was 1.6 cm^2^, and the error rate was 9.5% (Table 2).

### 3.2. Effects on Foot Structures of Different Push-Off Angles

**Figure 6:**
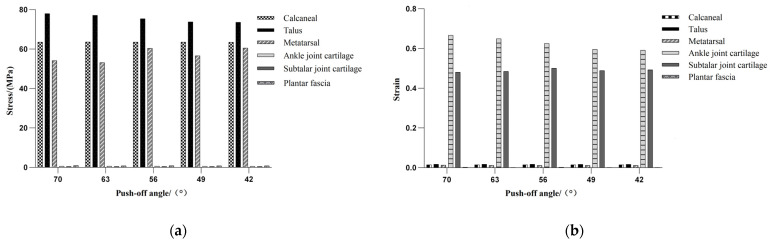
Stress and strain comparison chart. (**a**) Stress of the foot; (**b**) strain of the foot.

At the same push-off angle in speed skating, the stress on the talus was the highest, followed by the stress on the calcaneus, and the stress on the articular cartilage was the lowest. In addition, the strain on the ankle joint cartilage was the highest, while the strain on the subtalar joint cartilage was the second highest and the strain on the plantar fascia was the lowest (Table 3, Figure 6).

**Figure 7:**
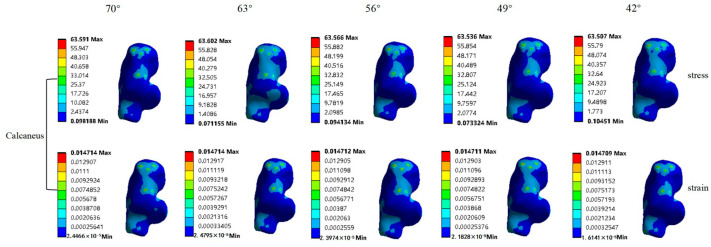

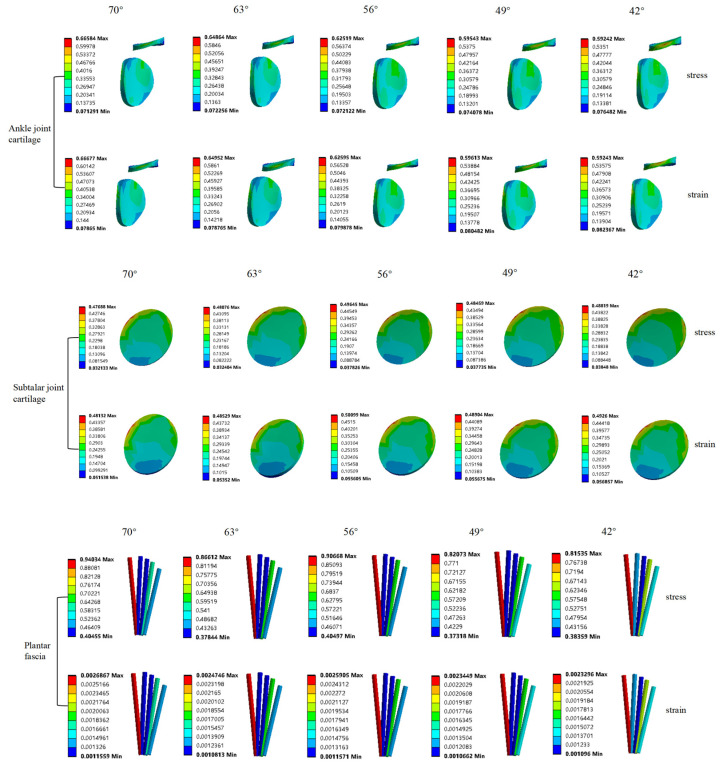
Stress and strain distributions for foot structures at different push-off angles.

During different stages of the speed-skating process, there were different changes in the stress and the strain on the foot bones, the articular cartilage and the plantar fascia. The stress and the strain on the calcaneus changed a little and were mainly concentrated on the medial side during speed skating. The stress on the talus illustrated an overall decreasing trend, with a decrease of 4.42 MPa from the pre-stroke to the post-stroke stages. Meanwhile, with the strain fluctuated less throughout the process, with the stress and the strain on the talus mainly concentrated in the posterior lower part near the heel. The stress on the metatarsal bones illustrated an overall increasing trend, with an increase of 7.392 MPa from the pre-stroke to the post-stroke stages, while there was little change in the strain. Furthermore, the stress and the strain on the metatarsal bones were mainly concentrated on the first and fourth metatarsal bones near the metatarsophalangeal joint. Both the stress and the strain on the ankle joint cartilage illustrated an overall decrease, with a decrease in stress of 0.074 MPa. Contrary to this, both the stress and the strain of the subtalar joint cartilage showed overall increasing trends with minimal fluctuations. The stress on the plantar fascia showed an overall decreasing trend, with a 0.074 MPa decrease in the pre-stroke stage, a 0.041 MPa increase in the mid-stroke stage and then a 0.086 MPa decrease again in the post-stroke stage, and the same trend can be seen in the strain (Figure 7).

## 4. Discussion

### 4.1. Validity of the FEM

In this study, a complete FEM of the foot and the speed skate was established based on CT scans and 3D laser-scanned images. The model was verified by comparing the peak pressure, contact area and pressure distribution results of the plantar and the bottom of the blade between the FEM prediction and in vivo measurements. The data show that the measured and simulated error rate of the peak pressure was less than 10%, and the stress was concentrated in the heel and forefoot positions, which was consistent with the findings of similar previous studies and further proved the validation of the FEM [36]. However, the values of the contact area of the bottom of the blade obtained by measuring and simulating were 16.8 cm^2^ and 15.2 cm^2^, respectively, which were larger than the actual contact area of the bottom of the blade. The reason for this is that a cushion layer of 2 mm of EVA was added between the speed skate and the support plate during the measurement and simulation, respectively, in order to avoid damage from the speed skate to the pressure test plate and to maintain consistency between the measured and simulated values. Despite the contact area values of the bottom of the blade increasing due to the wrapping of the soft EVA cushion, the error rate of the contact area between the measurement and simulation was less than 10%; thus, the FEM is valid.

### 4.2. Mechanical Response to Stress and Strain on the Foot Structure

The push-off angle is a key index for evaluating an athlete’s skating technique. In related research, the push-off angle was mainly analyzed via video recording, electromyography acquisition and model analysis. However, there are few studies on the internal mechanical response of the foot during speed skating, and it is impossible to directly reflect the technical mechanical characteristics of speed skating from the perspective of human body mechanics. The FEM established in this study provides a possibility for exploring the influence of the different push-off angles on the internal structure of the foot during speed skating.

A suitable load is beneficial to the growth and development of bones and fascia. However, an excessive load or frequent loads of a certain value can cause different degrees of damage, such as stress fractures or plantar fasciitis [18,19,20]. Abnormal stress in the foot and ankle is the main cause of injury in this area, and with greater stress comes a higher risk of injury [18,19,20]. In this study, the maximum stress values of the bones, articular cartilages and plantar fascia were all within their ultimate strengths at different push-off angles, resulting in low risks of fracture and the fascial rupture of the foot structure during speed skating [37]. However, it is worth noting that the maximum stress values of the talus, the calcaneus and the metatarsal bones were either close to exceeding or exceeded half of their ultimate strength during speed skating [37]. Moreover, in a competitive sport of the cyclical type, such as speed skating, frequent skating training and competition may increase the risk of injury through repeated frictional stresses on the bones and joints of the athletes’ feet and may even cause fatigue due to insufficient recovery [20]. From the strain perspective, excessive deformation may change the properties of the tissue material, thereby reducing its mechanical properties. When strain is excessive or not sufficiently rested, a stress response may occur, eventually leading to a stress fracture. In this study, the risk of injury of foot was low since the peak strain values of the calcaneus, the talus, the metatarsal bones and the plantar fascia were small and fluctuated less with the changes in the push-off angle, and the probability of abnormal tensile strain was low. In contrast, the strain peak values of the ankle joint cartilage and the subtalar joint cartilage were larger and fluctuated more with changes in the push-off angle. Such frequent changes and large strain values are likely to produce stress responses and cause a risk of fracture, eventually leading to a higher risk of fatigue or injury in the speed-skating process.

This study showed that the stress peaks of the calcaneus, the talus, the ankle joint cartilage and the plantar fascia were higher in the pre-stroke stage and lower in the post-stroke stage, but the stress peak of the metatarsal bones showed the opposite trend, which was partially consistent with the findings of previous studies on the correlation between skating techniques and plantar pressure distribution [38]. This study also has found that not all stresses and strains of the foot structures would increase as the push-off angle decreased. In speed skating, the center of gravity of the human body will change with the advance of the skating. In the pre-stroke stage, where the push-off angle was larger, the knee joint started to extend [39], and the biceps and hallux valgus muscles began to work [34]. Meanwhile, the push-off force from the legs was transmitted to the foot through the knee and ankle joints, where the talus, the plantar fascia and the ankle joint cartilage had the highest stress values. The reason is that after the contact between the speed skate and the surface of the ice was completed, the athlete’s center of gravity shifted from the sliding leg to the skating leg, and the muscle force was then transferred to the plantar so that the talus and the ankle joint cartilage at the rear part of the foot were the first to be squeezed. Therefore, the risk of injury to the talus, the ankle joint cartilage and the plantar fascia was higher in the pre-stroke stage of skating. In the mid-stroke stage, the push-off angle began to decrease, and the knee angle underwent a short reduction in the pre-lengthening-contraction effect of the muscles [34]. At this point, the talus and the plantar fascia remained at high stress levels, while the stresses of the metatarsal bones and the subtalar joint cartilage began to increase, suggesting that the force from the lower extremity was transmitted from the posterior to the anterior part of the foot in this stage. In the post-stroke stage of skating, with the further reduction in the push-off angle, the stress values of the talus, the plantar fascia and the ankle joint cartilage reached their lowest values, while the stress of the metatarsal bones was the highest. This indicated that the transfer of force from the posterior part of the foot to the anterior part of the foot was completed, resulting in a greater risk of injury for the metatarsal bones, which is also consistent with theories from the previous study [34] (Table 4). Based on the table, we could find that in different stages, the possibility of injuring the foot structure varies according to the knee angle and push-off angle.

In the skating process, the stress values of the calcaneus and the subtalar joint cartilage fluctuated less with changes in the push-off angle. A possible reason for this is that while the calcaneus and the subtalar joint cartilage were the main weight-bearing structures, they were less involved in the skating process at a certain angle. Consequently, their risk of injury was low. However, according to the concentration of stress on the calcaneus and the talus, the stress was concentrated in the upper part of the calcaneus and the lower part of the talus in skating with a certain push-off angle, also suggesting that the subtalar joint, where the calcaneus is connected to the talus, is also a more vulnerable area. Although the mechanical response mechanisms between different push-off angles and foot structures were analyzed in this study, according to the numerical changes in the stress and strain on the foot structures when the push-off angle changed, more in-depth studies are needed to determine the relationship between specific push-off angles and the risk of injury to the foot structure.

### 4.3. Limitations

In this study, there are several limitations that should be considered when dealing with FEM simulations in the future. Firstly, considering the efficiency and convergence of the calculation in the modeling process, certain simplifications were made to the structures of the speed-skate upper and the foot muscles which were still acceptable in other research studies. Although these simplifications caused some errors in the FEM, such possibly altering the loading conditions of the foot, they did not affect the final calculation results and conclusions [40]. With the development of medical instruments, we can build more accurate FEMs, such as by establishing detailed foot muscles and assigning appropriate material properties. Second, the simulations of the foot structures and sport performance were based on high-level athletes in this study, so the conclusion was targeted for the individual study of high-level athletes. Further work should be conducted to consider various patients, such as the general public. Third, the push-off force and the Achilles tendon force under the loading conditions of the FEM in this study were extracted from references, and only the push-off angle and the forces in certain directions were used. As we all know, a static simulation is not a complete representation of the movement of speed skating, and a dynamic simulation is more suitable.

### 4.4. Practical Applications

In the future, relevant research studies will be carried out to obtain the actual push-off force, and dynamic simulations of speed-skating movement should be considered to improve the accuracy of the FEM prediction so as to comprehensively study the continuous mechanical response of each structure of the foot with different push-off angles, further improving the performance of athletes.

## 5. Conclusions

In the present study, an FEM of a foot and a speed skate established was proved to be effective and can be used for further simulation research. The results indicate that not all foot structures have an increased risk of injury as the push-off angle decreases when the range of the push-off angle was 70–42°. The risks of injury to the talus, the ankle joint cartilage and the plantar fascia were higher with a greater push-off angle, while the risk to the metatarsals was higher with a smaller push-off angle. Therefore, we recommend that the push-off angle may be related to partial foot injury, and a change in the push-off angle could cause damage to different structures. The FEM of a foot and a speed skate developed in this study provides the possibility of further determining the relationship between foot injuries and skating technique.

## Figures and Tables

**Table 1:** Material properties of the finite element model.

Component	Unit Type	Modulus of Elasticity (MPa)	Poisson’s Ratio	Density (kg·m^−3^)	Cross-Section (mm^−2^)
Bone	SOLID187	7300	0.3	1500	--
Cartilage	SOLID187	1	0.4	--	--
Soft Tissue	SOLID187	1.15	0.49	937	--
Ligaments	SPRINGS	260	--	937	18.4
Plantar fascia	LINK180	350	--	937	290.7
Upper	SOLID187	80	0.45	476	5.01
Shoe upper lining	SOLID187	23.25	0.45	613	20.38
Knife rest	SOLID187	79,000	0.33	280	--
Ice Blade	SOLID187	210,000	0.30	7850	--
Ice surface	SOLID187	560	0.35	915	--
Support plate	SOLID187	17,000	0.1	5000	--
EVA cushion	SOLID187	4.25	0.45	--	--

**Table 2:** The results of the validation.

Component	Plantar		Bottom of the Blade	
	Measurement	Simulation	Measurement	Simulation
Peak pressure/kPa	220	200	496	449
Contact area/cm^2^	106.8	114.9	16.8	15.2

**Table 3:** Stress and strain results for the foot at different push-off angles.

Push-Off Angle	70°		63°		56°		49°		42°	
Component	Stress/MPa	Strain	Stress/MPa	Strain	Stress/MPa	Strain	Stress/MPa	Strain	Stress/MPa	Strain
Calcaneus	63.591	0.015	63.602	0.015	63.566	0.015	63.536	0.015	63.507	0.015
Talus	78.036	0.018	77.197	0.018	75.411	0.018	73.823	0.017	73.616	0.017
Metatarsal	54.167	0.013	53.154	0.012	60.441	0.012	56.617	0.012	60.546	0.012
Ankle joint cartilage	0.666	0.667	0.649	0.650	0.625	0.626	0.595	0.596	0.592	0.592
Subtalar joint cartilage	0.477	0.481	0.481	0.485	0.496	0.501	0.485	0.489	0.488	0.493
Plantar fascia	0.940	0.003	0.866	0.002	0.907	0.003	0.821	0.002	0.815	0.002

**Table 4:** Analysis of speed skating.

Stage	Knee Angle	Push-Off Angle	Injured Structure of Possibility
Pre-stroke	↑	↓	Talus, ankle joint cartilage, plantar fascia
Mid-stroke	↓	↓	Talus, plantar fascia
Post-stroke	↑	↓	Metatarsal bones

## Data Availability

The data presented in this study are available upon request from the corresponding author.
